# Effects of the LC mobile phase in vacuum differential mobility spectrometry-mass spectrometry for the selective analysis of antidepressant drugs in human plasma

**DOI:** 10.1007/s00216-022-04276-0

**Published:** 2022-08-17

**Authors:** Maria Fernanda Cifuentes Girard, Patrick Knight, Roger Giles, Gérard Hopfgartner

**Affiliations:** 1 Life Sciences Mass Spectrometry, Department of Inorganic and Analytical Chemistry, University of Geneva, 24 Quai Ernest Ansermet, CH-1211 Geneva 4, Switzerland; 2 Shimadzu Research Laboratory, Wharfside, Trafford Wharf Road, Manchester, M17 1GP UK

**Keywords:** Isobaric drugs, Antidepressants, Quantification, Differential mobility spectrometry, FAIMS, Trap-elute LC, Plasma

## Abstract

The effect of LC mobile phase composition and flow rate (2–50 µL/min) on mobility behavior in vacuum differential mobility spectrometry (vDMS) was investigated for electrosprayed isobaric antidepressant drugs (AD); amitriptyline, maprotiline, venlafaxine; and structurally related antidepressants nortriptyline, imipramine, and desipramine. While at 2 µL/min, no difference in compensation voltage was observed with methanol and acetonitrile, at 50 µL/min, acetonitrile used for LC elution of analytes enabled the selectivity of the mobility separation to be improved. An accurate and sensitive method could be developed for the quantification of six AD drugs in human plasma using trap/elute micro-LC setup hyphenated to vDMS with mass spectrometric detection in the selected ion monitoring mode. The assay was found to be linear over three orders of magnitude, and the limit of quantification was of 25 ng/mL for all analytes. The LC-vDMS-SIM/MS method was compared to a LC-MRM/MS method, and in both cases, inter-assay precisions were lower than 12.5 and accuracies were in the range 91.5–110%, but with a four times reduced analysis time (2 min) for the LC-vDMS-SIM/MS method. This work illustrates that with vDMS, the LC mobile phase composition can be used to tune the ion mobility separation and to improve assay selectivity without additional hardware.

**Graphical abstract:**

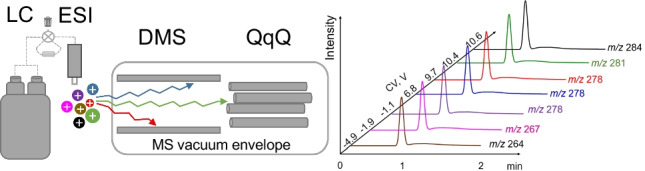

**Supplementary Information:**

The online version contains supplementary material available at 10.1007/s00216-022-04276-0.

## Introduction

Identification and quantification of antidepressants in urine and plasma is very important not only in forensic toxicology but also for therapeutic drug monitoring (TDM) to determine safety, tolerability, and efficacy of pharmacological treatment and pharmacokinetics [1]. Tricyclic antidepressant drugs are a group of the main category of antidepressant drugs (e.g., amitriptyline, desipramine, imipramine, and nortriptyline) which are commonly prescribed since 1950s to treat major depressive disorder. Many analytical procedures have been described to quantified antidepressant drugs in biological matrices down to the ng/mL level [2]. Gas chromatography with conventional and mass spectrometry (MS) detection [3], or liquid chromatography with diode array and mass spectrometry detection, being the most commonly used ones [4, 5]. Ion mobility spectrometry (IMS) [6] has also gained interest for the direct analysis of antidepressant drugs. In general IMS, separates ions in the gas phase based on their mobilities under the influence of electric field, either as a standalone system or coupled to mass spectrometry (IMS-MS) [7, 8].

Regarding applications to determine antidepressant drugs in diverse matrices, different ionization techniques have been coupled to ion mobility devices. Jafari et al. [9] described a method based on electrospray ionization-drift tube ion mobility spectrometry (ESI-DTIMS) for the simultaneous determination of desipramine and trimipramine in urine and plasma samples. Desorption electrospray ionization (DESI) was coupled to an ambient pressure drift time ion mobility time-of-flight mass spectrometer (DTIMS-TOFMS) for the direct analysis of active ingredients of pharmaceutical samples, including antidepressant drugs in tablets [10]. Corona discharge ionization (CD) was coupled to ion mobility for the determination of antidepressant drugs in urine [11], plasma [12], breast milk, and river water samples [13]. Piendl et al. [14] reported an online hyphenation of chip-based high-performance liquid chromatography (chip-HPLC) with ion mobility spectrometry (MS) to separate isobaric antidepressants.

Differential ion mobility or high field asymmetric waveform ion mobility exploits the electric field mobility where between two electrodes, an asymmetric waveform is applied orthogonal to the gas stream. A compensation voltage (CV) is required to compensate the drift of the ions towards the electrodes. By scanning the CV, ions with different mobilities can pass through the cell [15]. DMS is generally combined with triple quadrupole linear ion trap, quadrupole time of flight, or orbitrap and offers the possibility to add chemical modifiers (e.g., isopropanol, acetone, and toluene) to the transport gas flow to tune separation selectivity, and/or improve S/N ratio. Ruskic et al. [16] reported a liquid chromatography differential spectrometry mass spectrometry (LC-DMS × HRMS) approach using different modifiers for selectivity optimization for the analysis for isomeric sulfonamide drugs in human plasma. Werres et al. [17] described a study where they compared three different ion mobility spectrometry systems including traveling wave ion mobility spectrometer, a differential ion mobility spectrometer, and a differential mobility analyzer for the separation of small isomeric compounds. They concluded that IMS is a generic method for the separation of small isobaric and isomeric compounds regardless of the specific technique. DMS could also be successfully applied to separate isomers and conformers of doubly protonated cyclosporine analogues that are co-eluting using liquid chromatography [18]. Furthermore, DMS has been implemented to compensate for liquid chromatography selectivity loss in trap-elute approaches. Bravo-Veyrat et al. [19] reported a 1.5 min high-throughput a liquid chromatography differential spectrometry mass spectrometry using multiple reaction mode (LC-DMS-MRM/MS) method for determination of reduced and oxidized glutathione in human blood using ethanol as modifier. Up to 30 times higher sample throughput compared to LC–MS was reported using a chromatography-free DMS-MS method for the quantitation of seven urine metabolites in non-human primate urine [20]. The application of DMS-MS with chromatography was also demonstrated for fast quantitation of opioid isomers in human plasma using biocompatible solid-phase micro extraction (bio-SPME) and an open-port probe sampling interface [21].

While most DMS applications were developed on systems at atmospheric pressure, recently, a prototype DMS operating at low pressure regime 6–40 mbar, using a planar-gap stage within the MS instrument envelope extending the normalized electric field up to 543 Td, was reported by Shvartsburg et al. [22, 23]. Transit time below 30 ms makes vDMS compatible with LC-MRM/MS acquisition.

In the present work, we investigate first the effects of the LC mobile phase composition in vacuum differential mobility spectrometry coupled to triple quadrupole mass spectrometry (vDMS-MS) on resolution and selectivity for the separation of isobaric antidepressant drugs (i.e., amitriptyline, maprotiline, and venlafaxine). In addition, we describe the potential of vDMS as an additional separation dimension to reduce LC analysis time while maintaining good quantitative performance. A set of model compounds including isobaric antidepressants and structural related antidepressants nortriptyline, imipramine, and desipramine were used to develop an assay in human plasma using a short LC column operated in trap/elute mode (LC-vDMS-SIM/MS) and detection in the selected ion monitoring mode. The performance of this assay is compared to that of a liquid chromatography method using the multiple reaction monitoring mode (LC-MRM/MS).

## Experimental

### Materials and chemicals

Antidepressant standards, amitriptyline, amitriptyline-D_3_, maprotiline, venlafaxine, nortriptyline, nortriptyline-D_3_, imipramine, imipramine-D_3_, and desipramine were purchased from Sigma-Aldrich Co. (Buchs, Switzerland) (for structures, see Supplemental Info Figure S1). LC–MS grade acetonitrile were obtained from VWR Chemicals (Fontenay-sous-Bois, France) and Rathburn Chemicals (Scotland, UK), and methanol (Rathburn Chemicals), perchloric acid (70%), and acetic acid from Fischer Scientific AG (Reinach, Switzerland) and Rathburn Chemicals. UHPLC-MS grade water was from Huberlab (Aesch, Switzerland) and Rathburn Chemicals (Scotland, UK).

### Preparation of standard solutions

Stock solutions of antidepressants were prepared by dissolving each standard in MeOH at 1 mg/mL. The working solutions were diluted to the desired concentration in 50/50 MeOH/H_2_O, 0.1% formic acid.

### Preparation and treatment of plasma samples

Lyophilized citrate human plasma (P9523) was purchased from Sigma-Aldrich Co. (Buchs, Switzerland) and used for the LC-vDMS-SIM/MS method while heparin plasma generated from anonymized human blood donors (Centre de Transfusion Sanguine, HUG, Geneva, Switzerland) was used for the LC–MS method. For sample preparation, 75 μL of plasma was spiked with 25 μL of internal standards solution (2400 ng/mL including amitriptyline-D3, nortriptyline-D_3_, desipramine-D_3_, and imipramine-D_3_ for LC–MS method or 2400 ng/mL imipramine-D3 for LC-vDMS-SIM/MS method). For protein precipitation, 100 µL of 0.5 M perchloric acid was added to the mixture, vortexed, and centrifuged at 13,000 rpm for 10 min. To a volume of 100 µL of supernatant, 100 µL of 0.5 M ammonium formate was added to adjust the pH.

### Calibration and quality control samples for assay validation

Calibration curves were spiked in pooled human plasma to have a final concentration of 25, 125, 400, 600, 1000, and 2500 ng/mL and internal standard concentration of 150 ng/mL. LLOQ, QCLow, QCMedium, and QCHigh were prepared independently at three concentrations (25, 80, 500, and 1500 ng/mL). Assay linearity, accuracy, precision, detection limit, and quantitation limit were evaluated, and five replicate analyses were performed during three non-consecutive days.

#### LC-vDMS-SIM/MS analysis

Liquid chromatography was performed on a Nexera Mikros (Shimadzu Corporation, Kyoto, Japan) composed of one LC-30AD pump, LC-Mikros microflow pump, a SIL-30AC autosampler, a 6-port switching valve FCV-32AH, and a CTO-Mikros column oven with UF-Link. The UHPLC system was coupled to a LCMS-8060 triple quadrupole mass spectrometer equipped with a micro-electrospray source and a prototype vDMS cell operating at a pressure of 33 mbar (Shimadzu Research Laboratory, UK). The operating gas flows were the following: nebulizing gas (NB) flow 1.5 L/min (N_2_), drying gas (DG) OFF, heating gas flow OFF (air), CID gas 17 kPa, interface voltage 2 kV, DL temperature 250 °C, and heat block temperature 400 °C. For trap-elute LC, an additional pump (LC-30AD) was used and a Luna Omega C18 column (0.5 × 20 mm, 5 µm 100A°) was mounted on a 6-port switching valve as illustrated in Figure S2. In a first step, analytes were retained onto the column (25 °C) in front-flush using a mixture of H_2_O/CH_3_CN (95/5; v/v), 0.1% formic acid at 100 μL/min. After 0.25 min, the valve was switched and the analytes eluted in back-flush mode to the mass spectrometer using 100% v/v CH_3_CN, 0.1% formic acid at 50 μL/min. The total run time was 2 min and injection volume of 1 μL. Data were acquired and processed using LabSolutions (version 5.99 SP2, Shimadzu Corporation, Kyoto, Japan). Table S1 summarizes the LC, MS, and vDMS optimized parameters for the analysis of the six antidepressant drugs, and the MS/MS spectra are presented in Figure S3 (Supplemental Info).

#### LC-MRM/MS analysis

Liquid chromatography (column and conditions were identical to that reported by Kirchherr et al. [24]) was performed on a Nexera UHPLC (Shimadzu Corporation, Kyoto, Japan) composed of one LC-30AD pump, a SIL-30AC autosampler, and a CTO-30A oven. The UHPLC system was coupled to a LCMS-8050 triple quadrupole mass spectrometer with ESI source (Shimadzu Corporation, Kyoto, Japan). The nebulizing gas flow was of 1.5 L/min (N_2_), the drying gas flow of 3.0 L/min (N_2_), and the heating gas flow of 3.0 L/min (air). Argon was used as CID gas 270 kPa. The interface voltage was of 2 kV, the DL temperature of 200 °C, and the heat block temperature of 400 °C. The analytical column was a Chromolith Speed ROD C18 column (4.6 × 50 mm, 5 μm, Merk). The column temperature was set at 24 °C. Mobile phase A was 5 mM acetic acid in water and mobile phase B methanol. The gradient started at 20% B, and after 1 min, the gradient was linearly increase to 70% B in 3 min and kept constant for 1 min (70% B) followed by re-equilibration time of 3 min (20% B). The total LC run time was 8 min. The flow rate was set at 1.0 mL/min and injection volume to 10 μL. Data were acquired and processed using LabSolutions (version 5.99 SP2, Shimadzu Corporation, Kyoto, Japan). Table S1 summarizes the LC, MS, and vDMS optimized parameters for the analysis of the six antidepressant drugs.

## Results and discussion

### vDMS optimization

**Fig. 1:**
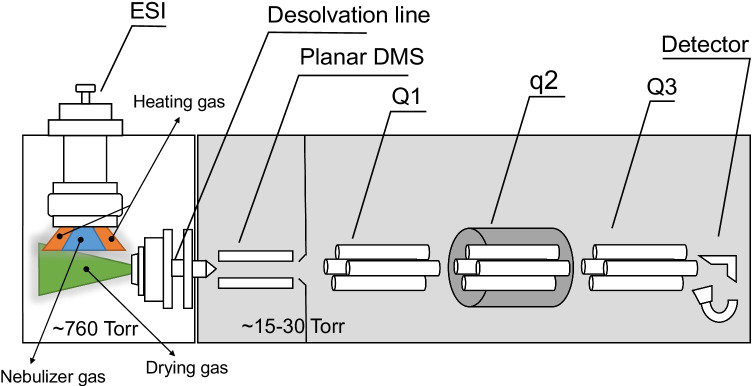
Schematic representation of the vacuum differential ion mobility mounted in the triple quadrupole

The prototype vDMS consists of a planar cell [22] is inserted into the vacuum manifold of the triple quadrupole and operated at a pressure 20–40 mbar (N_2_) as shown in Fig. 1. The dispersion plot (CV versus separation voltage (SV)) were recorded for three isobaric antidepressants (amitriptyline, maprotiline, and venlafaxine at *m/z* 278.2) electrosprayed by infusion at 8 µL/min.

The SV and CV values were scanned from 0 to 223 Td (SV) and 11.1 to − 1.9 Td (CV) (steps of 6 Td and 0.04 Td, respectively), and peak capacity (PC) and resolution (Rs) were calculated according to Eqs. (1) and (2) [15]:

1 $$PC={CV}_{\mathrm{max}}-{CV}_{\mathrm{min}}/1.7 \times {FWHM}_{\mathrm{averaged}}$$

2 $$Rs=1.18\times [({CV}_{1}-{CV}_{2})/{FWHM}_{1}-{FWHM}_{2}]$$

**Fig. 2:**
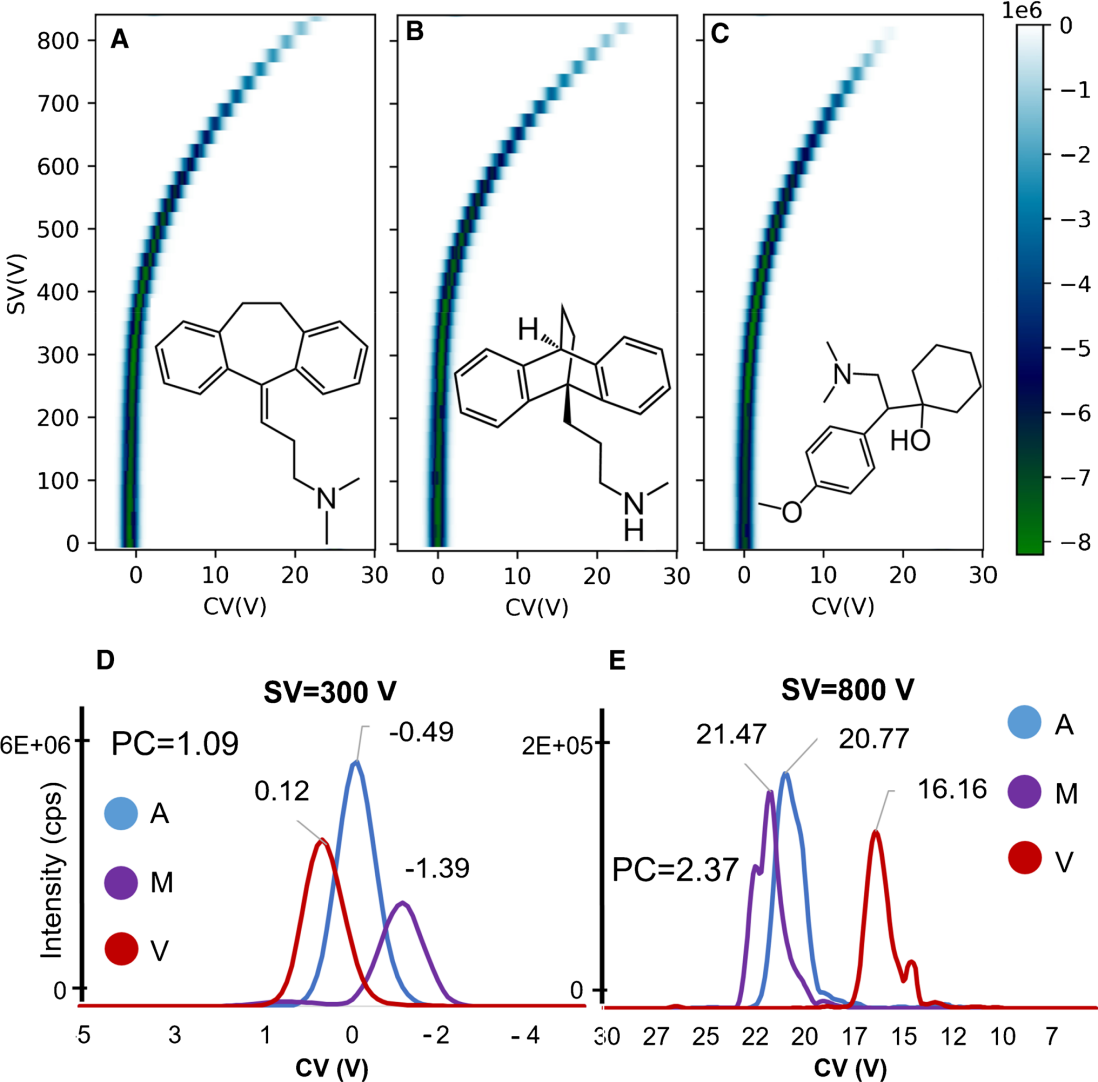
Dispersion plots (CV versus SV) with signal intensities of amitriptyline (**A**), maprotiline (**B**), and venlafaxine (**C**) in SIM mode. vDMS was used in scan mode (DV ramp from 0 to 900 V and CV stepped by 0.2 V). Overlaid compensation voltage plots for the three isobaric antidepressant drugs at SV of 300 (**D**) and 800 V (**E**). DMS cell temperature was of 60 °C, pressure 33 mbar, and nitrogen as a drift gas. The analytes were infused at 500 ng/mL at a flow rate of 8 μL/min (50/50 H_2_O/CH_3_CN 0.1% FA)

PC is calculated over a set of analytes where CV is the compensation voltage and *FWHM*_averaged_ is the full width at half maximum averaged of all peaks. The *Rs* is calculated between two isomeric peaks. Compared to a SV of 300 V, at 800 V, the analytes CV are positively shifted by about 15 to 20 V, as typically observed for type C behavior resulting from hard-sphere interactions [15]. At SV of 760 V, the peak capacity improved by a factor of 2 with a decrease of absolute sensitivity of about 20 times, compared to SV of 300 V (Fig. 2).

**Fig. 3:**
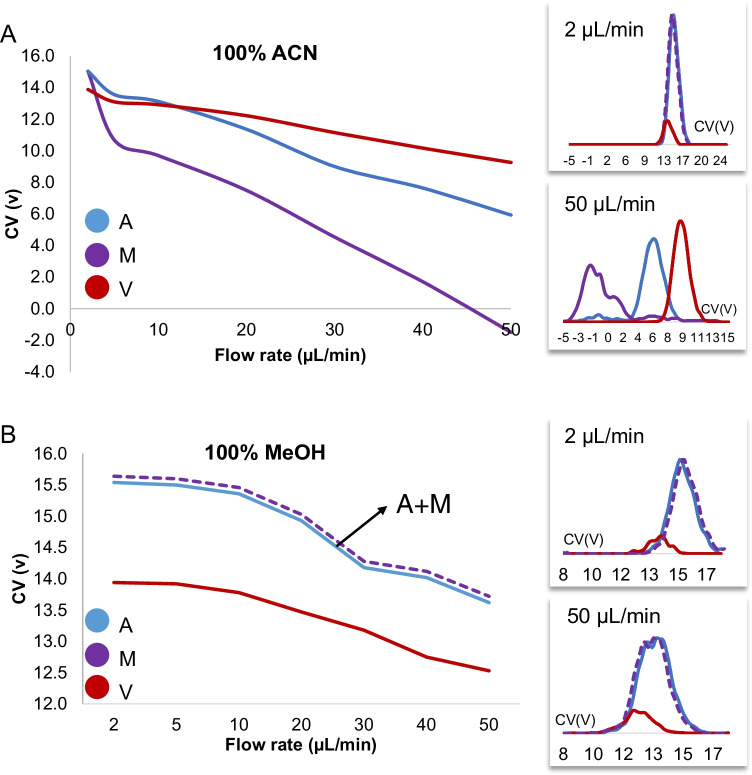
Effect of the increase flowrate, from 2 to 50 μL/min, in resolution and peak capacity of 100% acetonitrile, 0.1% FA (**A**) and 100% methanol, 0.1% FA (**B**) infusion mobile phase. The SV was of 800 V and CV steps of 0.2 V

This gain in peak capacity is not sufficient to obtain a baseline separation of the isobaric compounds, even at high E/N of 198 Td. In general, at fixed flow rates, the recorded relative standard deviations (RSD) of the CV were found to be less than 15% inter-day and intra-days (data not shown). However, it was observed that infusion or LC flow rates > 10 μL/min and mobile phase composition did affect the CV, which were shifted to more negative values suggesting a type A or B behavior. Type A behavior results from a repetitive clustering and declustering of an ion with polar neutral species in the transport gas. Therefore, the effect of infusion flow rates (from 2 to 50 μL/min) and mobile phase composition (100% methanol and acetonitrile with 0.1% FA) on resolution were investigated for amitriptyline, maprotiline, and venlafaxine and are presented in Fig. 3. At 2 µL/min, all analytes showed CV around 13–17 V while at 50 μL/min with 100% acetonitrile with 0.1% FA as mobile phase, all analytes were shifted to more negative CV values by about 10 to 20 V and were almost baseline separated. For methanol with 0.1% FA, only a small negative shift of 3–4 V was observed.

Compared to methanol, acetonitrile solutions provided the best resolution *RS*_A_ = 2.36, *RS*_M_ = 4.13, and *RS*_V_ = 1.70, and the best peak capacity was also observed for acetonitrile *PC* = 3.50, compared to methanol (*PC* = 0.75) (Supplemental Info Figure S4). The use of polar protic solvent such as methanol broadens peaks leading to lower resolution of the isobaric antidepressant compounds (*RS*_A_ = 0.11, *RS*_M_ = 0.93, *RS*_V_ = 0.77) compared to a polar aprotic solvent such as acetonitrile. The separation performance in ambient-pressure DMS is influenced by several parameters, such as the SV, the drift gas type (e.g., nitrogen), the cell geometry, and the temperature. With the vDMS, pure nitrogen is introduced in the cell but at higher infusion or LC flow rate (50 μL/min), a CV shift to more negative values and better resolution can be observed similar to the case where modifiers are introduced in the drift gas of atmospheric pressure DMS cell. One can postulate that a small fraction of the solvent is getting into the mobility cell through the interface and is playing a role in the clustering-declustering mechanism even at very low concentration as reported in atmospheric pressure DMS, in which pure or mixtures of organic solvents are introduced by a syringe or peristaltic pump in the DMS nitrogen carrier gas at fixed concentration between 0.1 and 3% (mole ratio N_2_/solvent) [25].

### Trap/elute setup with short LC column using vDMS for the analysis of antidepressant drugs in human plasma

The simplest way to significantly decrease the analysis time of a LC-SRM/MS assay is to reduce chromatographic separation using a shorter column (typically 10–20 mm length). This can become problematic with regard to selectivity considering co-eluting compounds in particular when isobaric or isomeric drugs are analyzed. Nortriptyline, desipramine, and imipramine have different molecular weights and can be distinguished by their specific SIM traces or MRM transitions even when they co-elute. Amitriptyline and maprotiline are isomers, and isobars to venlafaxine, and can be differentiated by specific MS/MS mass fragments (*m/z* 278 > *m/z* 233, *m/z* 278 > *m/z* 250, and *m/z* 278 > *m/z* 58 see Figure S3, Supplemental Info) but at the cost of assay performance. The introduction of DMS as second orthogonal separation dimension to LC–MS allows separation of co-eluting analytes in LC without any change in analysis time. The temperature of the ion mobility cell was 60 °C, and the N_2_ pressure was 33 mbar. Several vDMS parameters were optimized, including flow rate (2–50 μL/min), organic solvent (methanol, acetonitrile), SV, and CV. For further analysis, an SV of 760 V (E/N 188 Td) was used, and CV values for each analyte are summarized in Table S1. Under the conditions investigated, not all analytes were baseline separated using vDMS such as imipramine and venlafaxine, but the three isobaric compounds showed different CV. The use of vDMS also significantly reduced the background noise which improves the S/N in SIM mode.

Sensitivity of an assay also depends on the injection volume, which can be critical with small i.d. columns (≤ 1 mm). In the LC-vDMS-SIM/MS method, the sample injection volume was reduced to 1 µL. In the trap-elute LC setup, a short column (20 mm) is mounted on a six-port switching valve. In a first step after injection, the analytes are retained onto the column while salts and polar endogenous compounds are washed out. In a second step, a ballistic organic elution (100% CH_3_CN, 0.1% FA) is applied in front-flush or back-flush mode, and the analytes are eluted to the mass spectrometer. These conditions were found best for good ESI sensitivity and vDMS separation. In the present case, the sample is loaded in front-flush with a high aqueous mobile phase (CH_3_CN/H_2_O 5/95 v/v) at 100 μL/min into the column, and perchloric acid/ammonium formate and other salts are washed out while the antidepressant drugs are retained. After 0.25 min, the valve is switched to allow elution of the retained analytes in back-flush mode with 100% v/v CH_3_CN, 0.1% FA at 50 μL/min. The trap/elute setup enables (i) to select the best solvent conditions (composition and injection volume) for pre-concentrating the analytes, (ii) to wash interferences, and (iii) to select the elution solvent with best DMS performance.

**Fig. 4:**
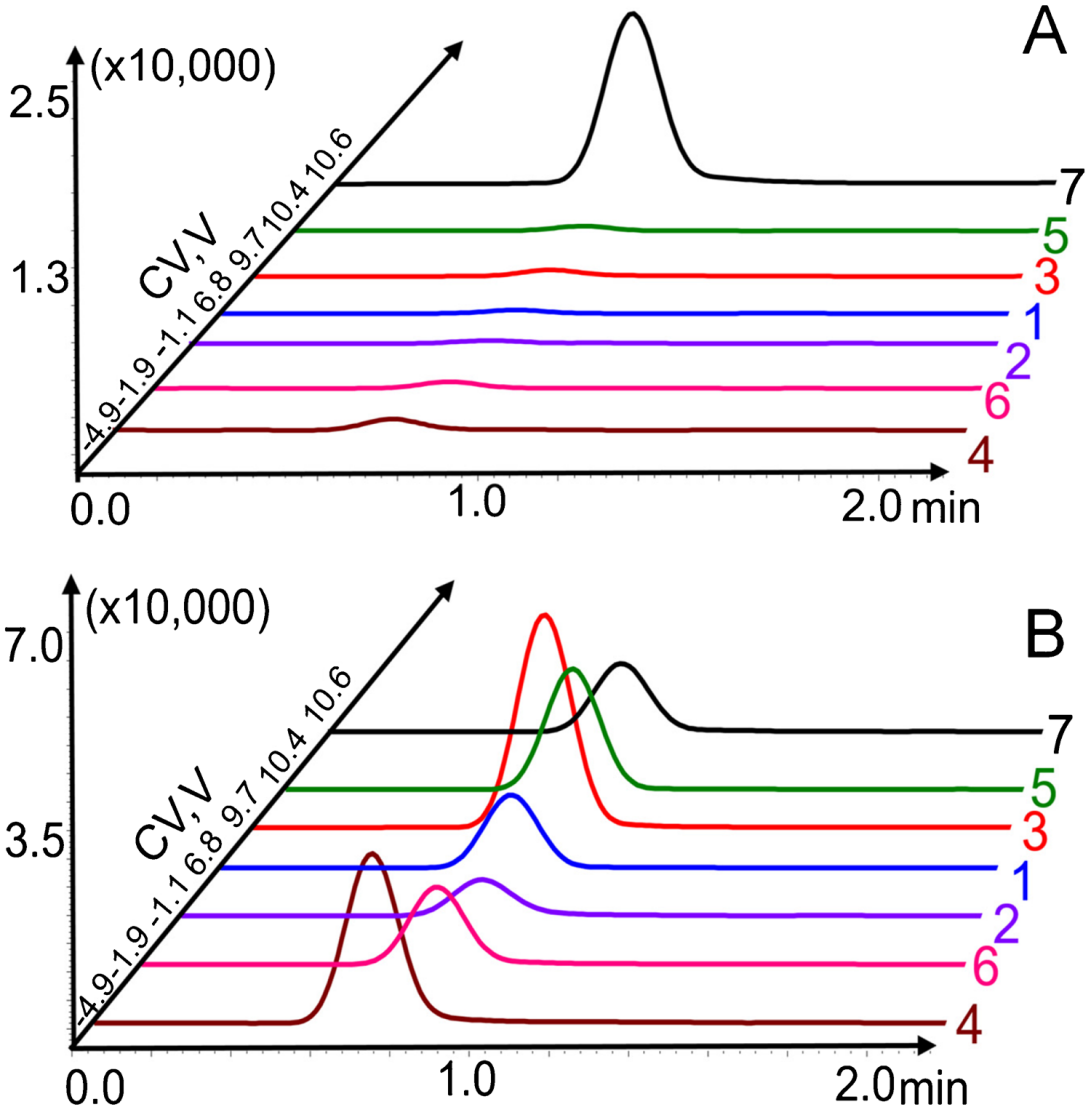
Representative XIC chromatograms by LC-DMS-SIM/MS **A** human plasma blank spiked with IS 150 ng/mL and **B** spiked human plasma at 75 ng/mL (IS 150 ng/mL). (1) Amitriptyline, (2) maprotiline, (3) venlafaxine, (4) nortriptyline, (5) imipramine, (6) desipramine, (7) IS, imipramine-D3

Representative XIC chromatograms of a blank human plasma and a human plasma spiked at 75 ng/mL with all analytes are presented in Fig. 4A  and B (more detailed view in Supplemental Info Figure S7). The combination with vDMS enables analyte baseline separation based on mobility in the gas phase at high/low field for a rapid quantitative analysis (less than 2 min), improving the S/N ratio and selectivity of the isobaric antidepressant drugs enabling detection in SIM mode.

**Fig. 5:**
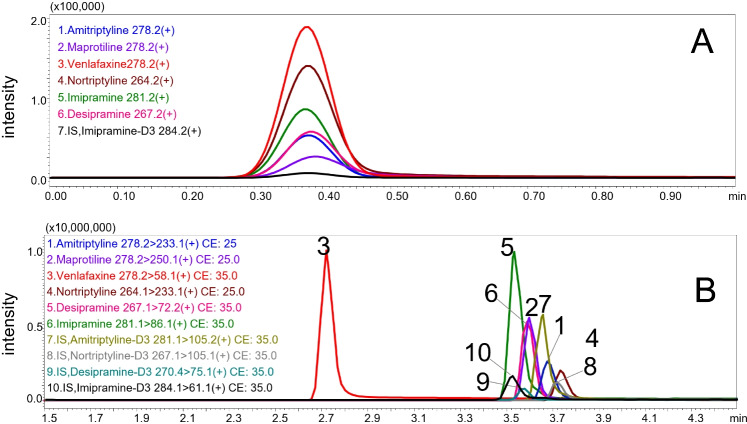
Representative zoomed chromatograms of human plasma with 400 ng/mL antidepressant drugs (IS 150 ng/mL) **A** LC-DMS-SIM/MS (2 min) and **B** LC-MRM/MS (8 min), individuals XIC are presented in Figure S6

To evaluate the performance of the short LC-vDMS-SIM/MS method, calibration and quality control samples (QCs) were prepared in human plasma. Accuracy, precision, linearity, and sensitivity were determined and compared to a standard LC-MRM/MS method. The analysis time of the LC-MRM/MS method was of 8 min using four isotopically labelled internal standards, and a representative chromatogram is shown in Figs. 5B and S6. In LC–MS analysis, to compensate matrix effects and for accurate quantification, the use of isotopically labelled internal standards is highly recommended for each analyte, and individual deuterated IS were used for amitriptyline, nortriptyline, desipramine, and imipramine. For the quantification of venlafaxine and maprotiline, amitriptyline-D3 was used as IS. In the trap-elute LC-vDMS-SIM/MS method, all analytes co-elute, so only one IS (imipramine-D3) is required which simplifies method development and reduces cost.

For both methods and all the analytes (including the LLOQ), inter-assay precision based on QC samples was lower than 12.5% and inter-assay accuracy was in the range 86.1–111% for the concentration range 25 to 2500 ng/mL and are summarized in Table 1, and in Supplemental Info Figures S5 and S6. These results show that LC-vDMS-SIM/MS method enables analysis of four times more samples in the same time period using only one IS and similar performance (linearity, accuracy, precision, limit of quantification) compared to the LC-MRM/MS (note that the injection volume in LC-vDMS-SIM/MS was 1/10 of that in the LC-MRM/MS method). The present assay demonstrated that vDMS is also a practical approach in bioanalysis as atmospheric pressure DMS. DMS offers an additional selectivity dimension for the development of quantitative assay. The purpose of the present work was not to achieve better sensitivity compared to the conventional LC–MS/MS assay but the possibility to significantly reduce LC analysis time.

**Table 1:** Inter-assay precision and accuracy for quality control (QC) of plasma samples spiked with antidepressant drugs. *AMI*, amitriptyline; *MAP*, maprotiline; *VEN*, venlafaxine; *NOR*, nortriptyline; *IMI*, imipramine; *DES*, desipramine for different methods applied. *LLOQ* lower limit of quantification, *LQC* low QC, *MQC* medium QC, *HQC* high QC

QC level	Method		AMI	MAP	VEN	NOR	IMI	DES
LLOQ (25 ng/mL)	LC-vDMS-MS (SIM)	Accuracy (%)	102	91.8	104	100	107	97.3
		Precision (%)	10.4	8.75	12.9	8.91	10.2	9.94
		S/N	52	41	350	253	243	117
	LC–MS (MRM)	Accuracy (%)	110	100	114	101	110	108
		Precision (%)	6.12	8.59	6.58	2.85	1.97	4.68
		S/N	327	702	50	384	436	1637
LQC (80 ng/mL)	LC-vDMS-MS (SIM)	Accuracy (%)	100	95.4	93.4	99.3	106	86.1
		Precision (%)	8.12	6.71	7.23	12.5	11.3	5.63
		S/N	424	178	1492	694	622	367
	LC–MS (MRM)	Accuracy (%)	93.3	89.3	87.4	87.0	89.8	92.0
		Precision (%)	5.94	4.84	4.28	9.45	7.13	3.67
		S/N	594	1296	120	1096	2157	2343
MQC (50 ng/mL)	LC-vDMS-MS (SIM)	Accuracy (%)	98.5	93.9	94.8	93.3	93.6	98.4
		Precision (%)	7.44	10.8	9.59	8.46	9.12	8.15
		S/N	1964	448	6551	2135	2868	901
	LC–MS (MRM)	Accuracy (%)	99.5	108	92.3	96.6	111	104
		Precision (%)	7.80	6.43	4.29	5.66	5.85	10.9
		S/N	8018	12,012	402	8805	24,475	17,128
HQC (1500 ng/mL)	LC-vDMS-MS (SIM)	Accuracy (%)	101	94.1	93.7	96.5	95.3	92.0
		Precision (%)	11.4	9.57	7.02	6.35	9.17	9.21
		S/N	2797	898	6702	3585	3946	1499
	LC–MS (MRM)	Accuracy (%)	91.5	97.3	93.8	94.9	93.4	95.0
		Precision (%)	9.29	6.15	9.29	7.33	4.94	6.39
		S/N	17,696	18,303	860	17,219	41,603	34,722

## Conclusions

In this study, we have investigated the effect of LC mobile phase on the differential mobility behavior of antidepressant drugs (AD) (including isomers and isobars) in a prototype vDMS planar cell inserted into the vacuum manifold of the triple quadrupole and operated at a pressure 20–40 mbar (N_2_). The results show that changing between the commonly used LC solvents, acetonitrile, and methanol, and altering the flow rate shift the CV and improve the separation selectivity of the analytes. In the case of AD drugs, the use of a polar aprotic organic solvent such as acetonitrile at higher flow rate (approx. 50 μl/min) enables separation of the isobaric analytes. A method combining a short C18 column with trap/elute LC setup hyphenated to vDMS and mass spectrometric detection in SIM mode was developed for the simultaneous quantification of isobaric antidepressants; amitriptyline, maprotiline, venlafaxine; and structural related antidepressants nortriptyline, imipramine, and desipramine with an LOQ of 25 ng/mL in human plasma. The LC-vDMS-SIM/MS method was compared to a LC-MRM/MS method, and in both cases, inter-assay precisions were lower than 12.5% but with a four times higher sample throughput for the vDMS method. The LC trap/elute DMS setup is not limited to antidepressant drugs and should be a practicable approach for different classes of compounds.

It is postulated that a small fraction of the LC organic solvent is transferred into the vDMS cell through the interface and acts as a modifier in a cluster/declustering mechanism without the use of additional hardware. This opens new analytical possibilities to improve vDMS performance based on LC mobile phase conditions either to significantly reduce analysis time and/or to remove potential interferences for improved quantification selectivity even in the selected ion monitoring mode. In the present work, the highest flow rate used was of 50 µL/min but preliminary investigations showed that higher flow rates up to 300 µL/min are also possible.

## Supplementary Information

Below is the link to the electronic supplementary material.

Supplementary file1 (PDF 2114 KB)

## References

[1] Schütze G, Schwarz MJ (2016). Therapeutic drug monitoring for individualised risk reduction in psychopharmacotherapy. TrAC Trends Anal Chem.

[2] Uddin MN, Samanidou VF, Papadoyannis IN (2011). Bio-sample preparation and analytical methods for the determination of tricyclic antidepressants. Bioanalysis.

[3] Manousi N, Samanidou VF (2019). Applications of gas chromatography for the analysis of tricyclic antidepressants in biological matrices. Separations..

[4] Esteve-Romero J, Albiol-Chiva J, Peris-Vicente J (2016). A review on development of analytical methods to determine monitorable drugs in serum and urine by micellar liquid chromatography using direct injection. Anal Chim Acta.

[5] Manousi N, Samanidou VF (2020). Recent advances in the HPLC analysis of tricyclic antidepressants in bio-samples. Mini-Rev Med Chem.

[6] Kirk AT, Bohnhorst A, Raddatz CR, Allers M, Zimmermann S (2019). Ultra-high-resolution ion mobility spectrometry-current instrumentation, limitations, and future developments. Anal Bioanal Chem.

[7] Cumeras R, Figueras E, Davis CE, Baumbach JI, Gracia I (2015). Review on ion mobility spectrometry. Part 1: current instrumentation. Analyst.

[8] Paglia G, Smith AJ, Astarita G (2021). Ion mobility mass spectrometry in the omics era: challenges and opportunities for metabolomics and lipidomics. Mass Spec Rev.

[9] Jafari MT, Saraji M, Sherafatmand H (2011). Electrospray ionization-ion mobility spectrometry as a detection system for three-phase hollow fiber microextraction technique and simultaneous determination of trimipramine and desipramine in urine and plasma samples. Anal Bioanal Chem.

[10] Roscioli KM, Tufariello JA, Zhang X, Li SX, Goetz GH, Cheng G (2014). Desorption electrospray ionization (DESI) with atmospheric pressure ion mobility spectrometry for drug detection. Analyst.

[11] Aladaghlo Z, Fakhari AR, Hasheminasab KS (2016). Application of electromembrane extraction followed by corona discharge ion mobility spectrometry analysis as a fast and sensitive technique for determination of tricyclic antidepressants in urine samples. Microchem J.

[12] Barati E, Alizadeh N (2020). Simultaneous determination of sertraline, imipramine and alprazolam in human plasma samples using headspace solid phase microextraction based on a nanostructured polypyrrole fiber coupled to ion mobility spectrometry. Anal Methods.

[13] Zamani F, Farajmand B, Yaftian MR (2020). Corona discharge ion mobility spectrometry combined by homogenizer assisted dispersive liquid-phase microextraction; a rapid and sensitive method for quantification of nortriptyline. Microchem J.

[14] Piendl SK, Raddatz C-R, Hartner NT, Thoben C, Warias R, Zimmermann S (2019). 2D in seconds: coupling of chip-HPLC with ion mobility spectrometry. Anal Chem.

[15] Schneider B, Nazarov E, Londry F, Vouros P, Covey T. Differential mobility spectrometry/mass spectrometry history, theory, design optimization, simulations, and applications: differential mobility spectrometry/mass spectrometry. Mass Spec Rev. 2015;35. 10.1002/mas.21453. 10.1002/mas.21453 25962527

[16] Ruskic D, Hopfgartner G (2019). Modifier selectivity effect on differential ion mobility resolution of isomeric drugs and multidimensional liquid chromatography ion mobility analysis. Anal Chem.

[17] Werres T, Leonhardt J, Jager M, Teutenberg T (2019). Critical comparison of liquid chromatography coupled to mass spectrometry and three different ion mobility spectrometry systems on their separation capability for small isomeric compounds (vol 82, pg 251, 2019). Chromatographia.

[18] Lam KHB, Le Blanc JCY, Campbell JL (2020). Separating isomers, conformers, and analogues of cyclosporin using differential mobility spectroscopy, mass spectrometry, and hydrogen-deuterium exchange. Anal Chem.

[19] Bravo-Veyrat S, Hopfgartner G (2018). High-throughput liquid chromatography differential mobility spectrometry mass spectrometry for bioanalysis: determination of reduced and oxidized form of glutathione in human blood. Anal Bioanal Chem.

[20] Chen Z, Coy S, Pannkuk EL, Laiakis EC, Fornace AJ, Vouros P (2018). Differential mobility spectrometry-mass spectrometry (DMS-MS) in radiation biodosimetry: rapid and high-throughput quantitation of multiple radiation biomarkers in nonhuman primate urine. J Am Soc Mass Spectr.

[21] Liu C, Gomez-Rios GA, Schneider BB, Le Blanc JCY, Reyes-Garces N, Arnold DW (2017). Fast quantitation of opioid isomers in human plasma by differential mobility spectrometry/mass spectrometry via SPME/open-port probe sampling interface. Anal Chim Acta.

[22] Shvartsburg AA, Haris A, Andrzejewski R, Entwistle A, Giles R (2018). Differential ion mobility separations in the low-pressure regime. Anal Chem.

[23] Andrzejewski R, Entwistle A, Giles R, Shvartsburg AA (2021). Ion mobility spectrometry of superheated macromolecules at electric fields up to 500 Td. Anal Chem.

[24] Kirchherr H, Kühn-Velten WN (2006). Quantitative determination of forty-eight antidepressants and antipsychotics in human serum by HPLC tandem mass spectrometry: a multi-level, single-sample approach. J Chromatrogr B.

[25] Ruskic D, Klont F, Hopfgartner G (2021). Clustering and nonclustering modifier mixtures in differential mobility spectrometry for multidimensional liquid chromatography ion mobility-mass spectrometry analysis. Anal Chem.

