# Supplementary material for: Effects of the LC mobile phase in vacuum differential mobility spectrometry-mass spectrometry for the selective analysis of antidepressant drugs in human plasma
Source: Anal Bioanal Chem. 2022 Aug 17;414(24):7243–52. doi: 10.1007/s00216-022-04276-0 (PMC9482904; doi:10.1007/s00216-022-04276-0)
Supplement: Supplementary file 1 — Supplementary file1 (PDF 2114 KB) [file 216_2022_4276_MOESM1_ESM.pdf]

Supplemental information

Effects of the LC mobile phase in Vacuum Differential Mobility Spectrometry-Mass Spectrometry for the Selective Analysis of Antidepressant Drugs in Human Plasma

Maria Fernanda Cifuentes Girard[1], Patrick Knight[2], Roger Giles[2], and Gérard Hopfgartner[1]*

1. Life Sciences Mass Spectrometry, Department of Inorganic and Analytical Chemistry, University of Geneva, 24 Quai Ernest Ansermet, CH-1211 Geneva 4, Switzerland
2. Shimadzu Research Laboratory, Wharfside, Trafford Wharf Road, Manchester M17 1GP, United Kingdom

* corresponding author e-mail: gerard.hopfgartner@unige.ch

Figure S1: Chemical structures of isobaric and structural related analytes. (1) amitriptyline (2) maprotiline (3) venlafaxine (4) nortriptyline (5) imipramine (6) desipramine.

Figure S2: Short LC-DMS-MS configuration.

Figure S3: MS/MS spectra of isobaric and related structural antidepressants drugs.

Figure S4: CV plots for amitriptyline, maprotiline and venlafaxine.

Figure S5: Calibration plots of plasma spiked with antidepressants drugs (25 to 2500 ng/mL).

Figure S6: Inter-assay accuracy at LLOQ level (25 ng/ml) of spiked antidepressant drugs in plasma samples

Figure S7: Representative zoomed chromatograms of human plasma for LC-MRM/MS method.

Table S1: MS conditions and RT for LC-DMS-SIM/MS and LC-MRM/MS.

Figure S1. Chemical structures of isobaric and structural related analytes. (1) amitriptyline (2) maprotiline (3) venlafaxine (4) nortriptyline (5) imipramine (6) desipramine. Red stars indicate atoms isotopically labeled for internal standards (D_3)

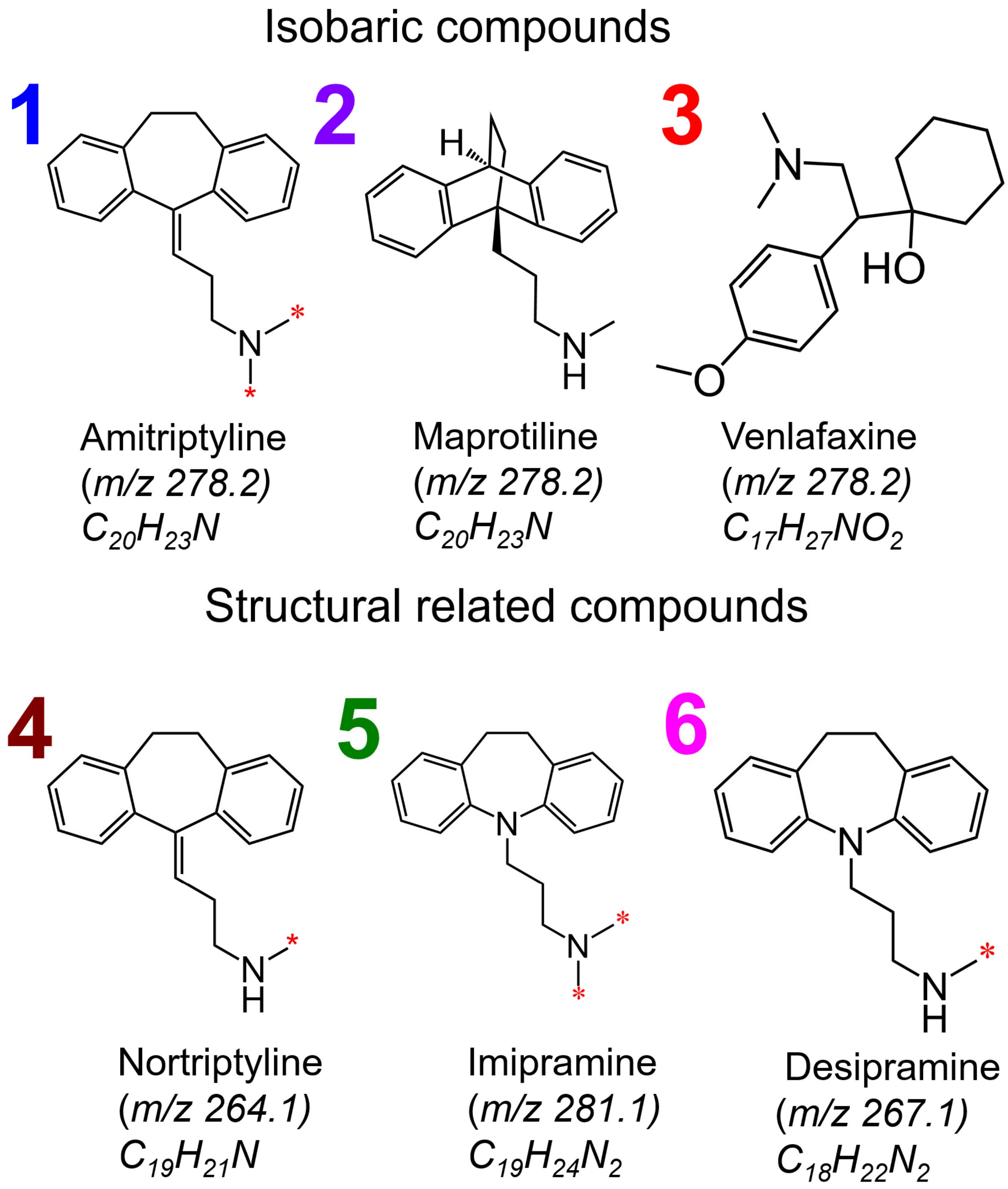

Figure S2. Product ion spectra of antidepressants drugs used in the study and acquired on LCMS-8050 (Shimadzu Corporation, Japan) at a collision energy of 35 eV.

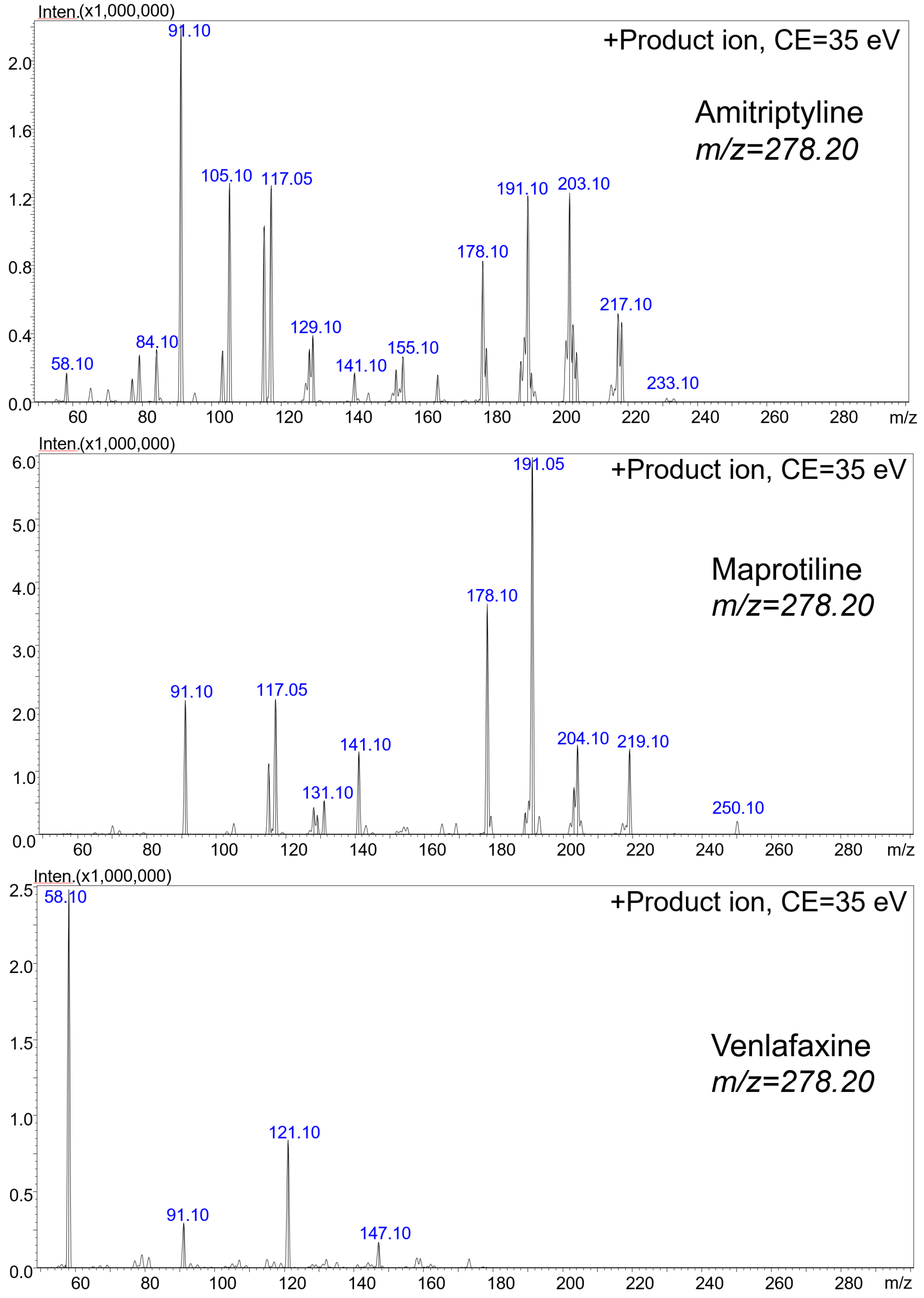

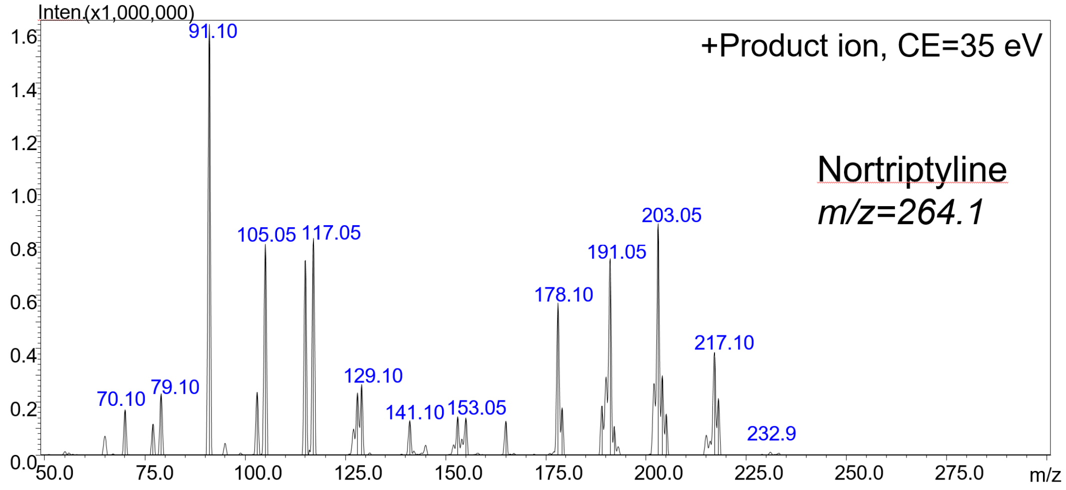
Inten.(x1,000,000)
+Product ion, CE=35 eV
Nortriptyline
m/z=264.1
91.10
105.05
117.05
203.05
191.05
178.10
217.10
70.10
79.10
129.10
141.10
153.05
232.9
m/z

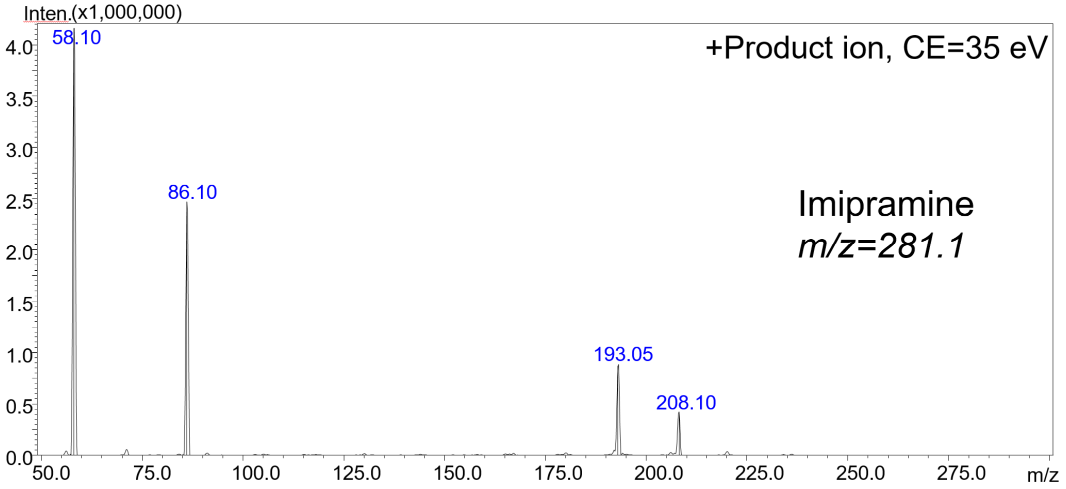
Inten.(x1,000,000)
+Product ion, CE=35 eV
Imipramine
m/z=281.1
58.10
86.10
193.05
208.10
m/z

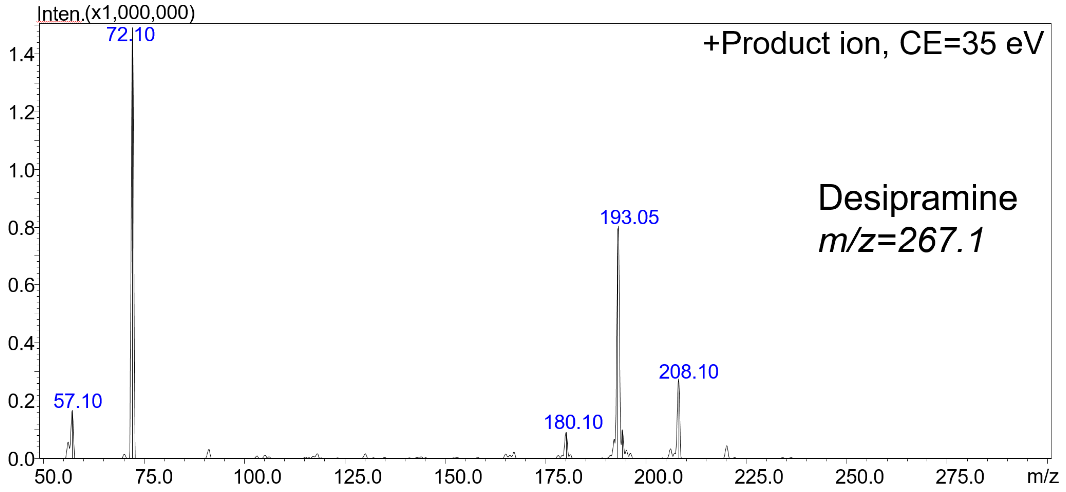
Inten.(x1,000,000)
+Product ion, CE=35 eV
Desipramine
m/z=267.1
72.10
193.05
208.10
57.10
180.10
m/z

Figure S3: Trap-elute LC-DMS-MS configuration. A short column Luna Omega C18 (20 x 0.5 mm, 5 µm 100A°) was used to analyzed the samples in 2 steps: front-flush injection (A) and back-flush elution of analytes (B) acquired in SIM mode of antidepressants drugs.

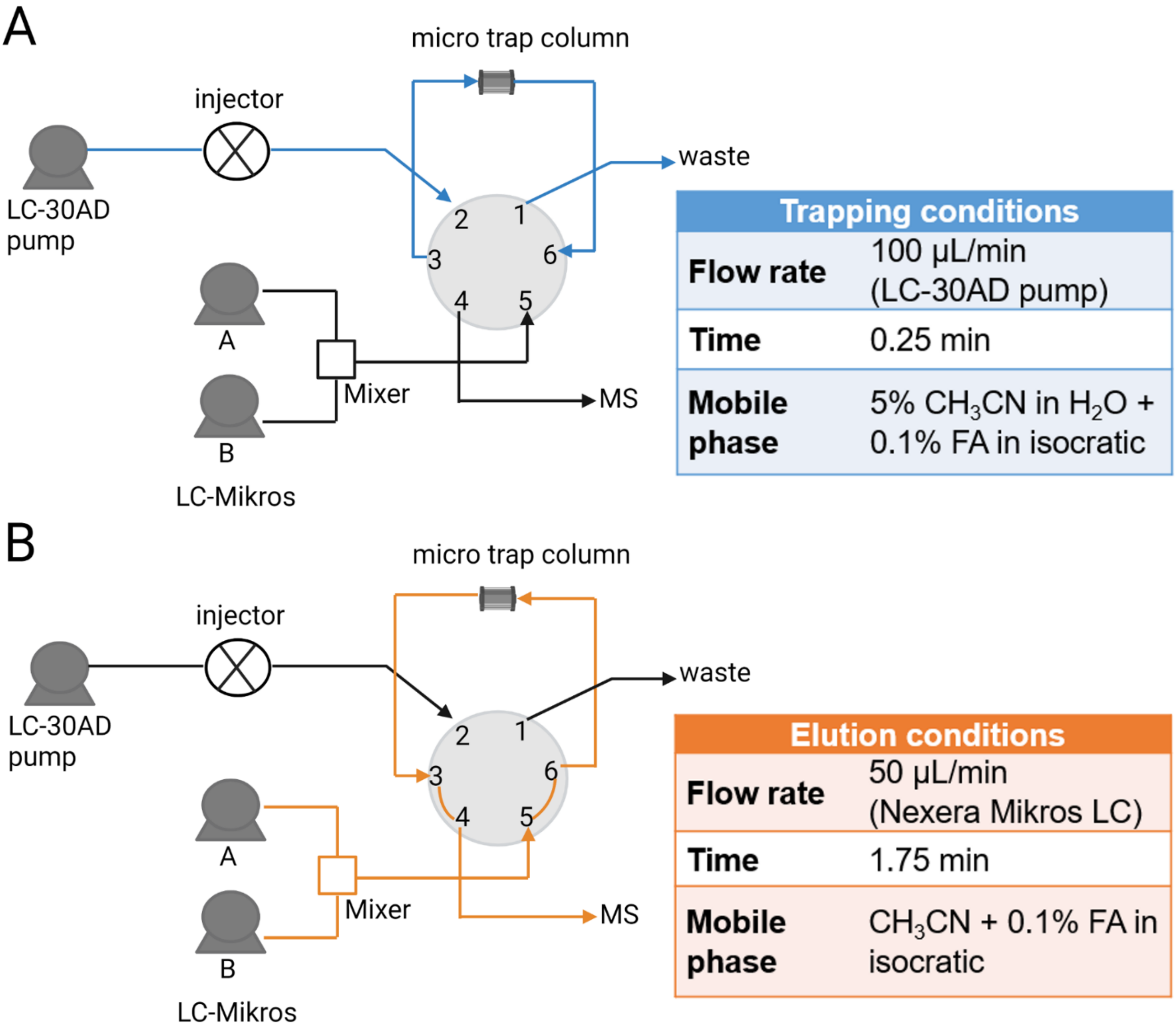

Figure S4: CV plots for amitriptyline, maprotiline and venlafaxine with A) 100 % acetonitrile 0.1 % FA, B) 100 % ethanol, 0.1 % FA. The analytes were infused at 500 ng/mL at a flow rate of 50 µL/min. The SV was of 800 V and CV steps of 0.2 V.

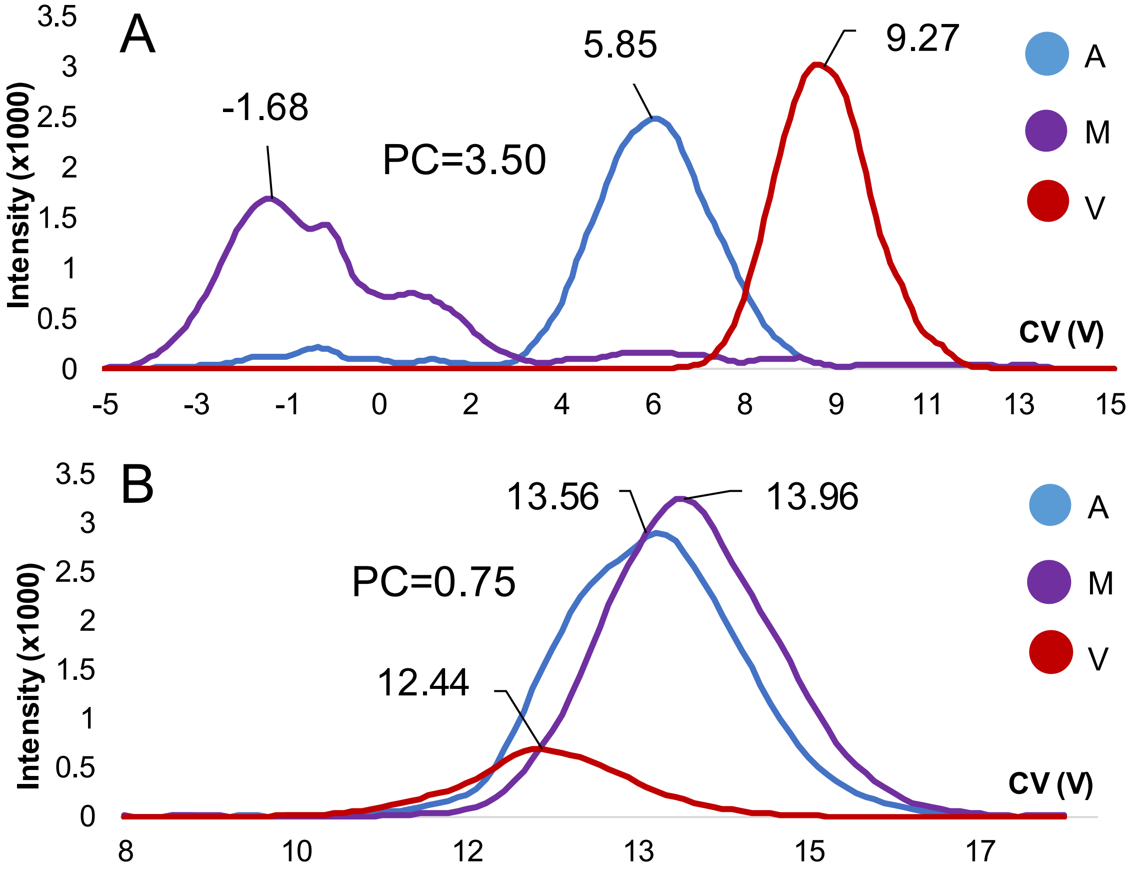

Figure S5. Calibration plots of plasma spiked with antidepressants drugs (25 to 2500 ng/mL). Triangles represents the accuracy QC levels (LLOQ, LQC, MQC, HQC). Samples were analyzed by the short LC-DMS-MS (acquired in SIM mode) highlighted in blue and LC-MS/MS (acquired in MRM mode) in green trace. Imipramine-D_3 was used as internal standard to correct matrix effect in short LC-DMS-MS in contrast to LC-MS was used the corresponding analog deuterium labelled compound. Amitriptyline-D_3 (for isobaric compounds), nortriptyline-D_3, desipramine-D_3 and imipramine-D_3.

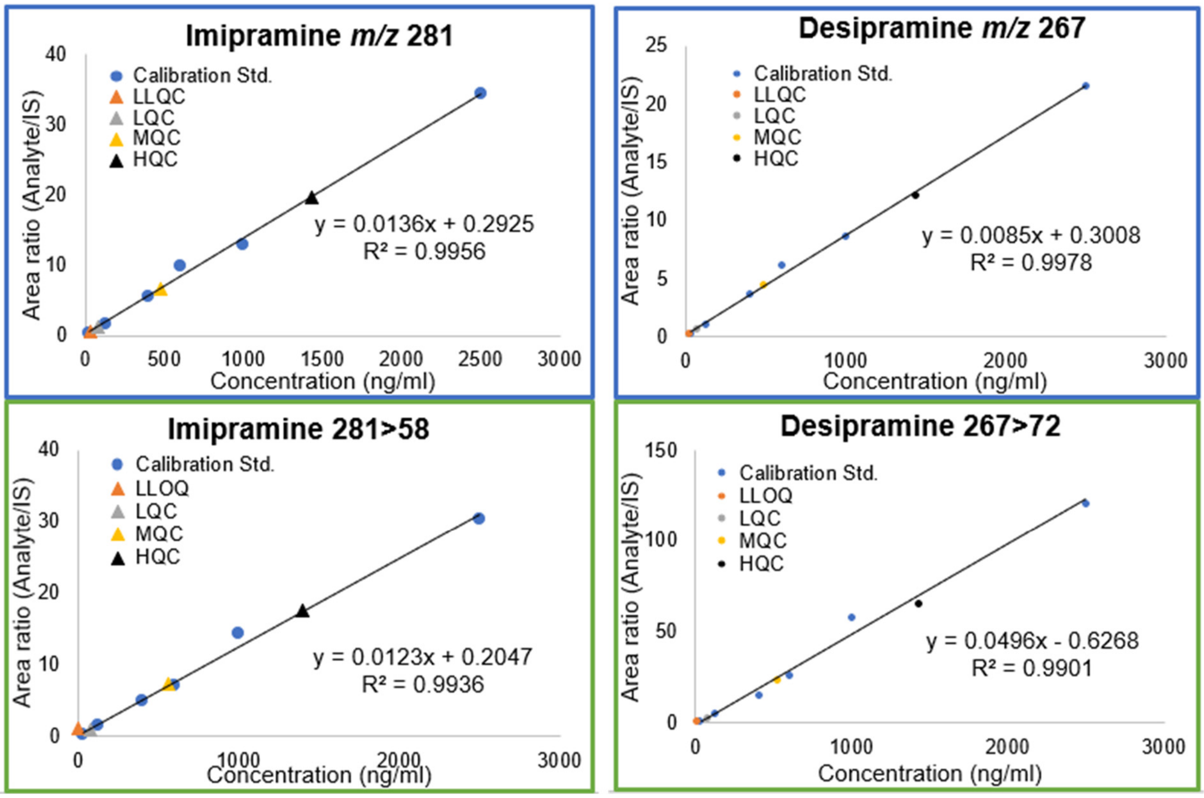

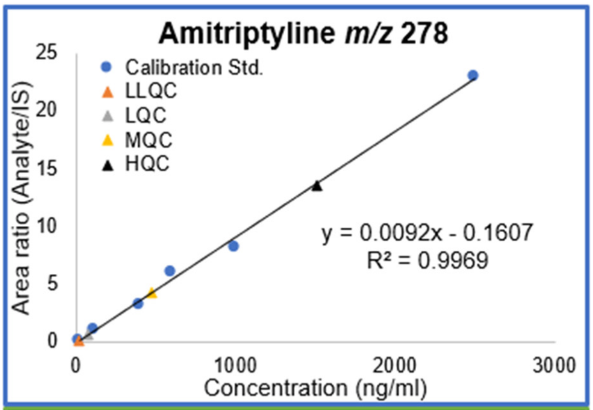
Amitriptyline m/z 278
Calibration Std.
LLQC
LQC
MQC
HQC
y = 0.0092x - 0.1607
R² = 0.9969
Area ratio (Analyte/IS)
Concentration (ng/ml)

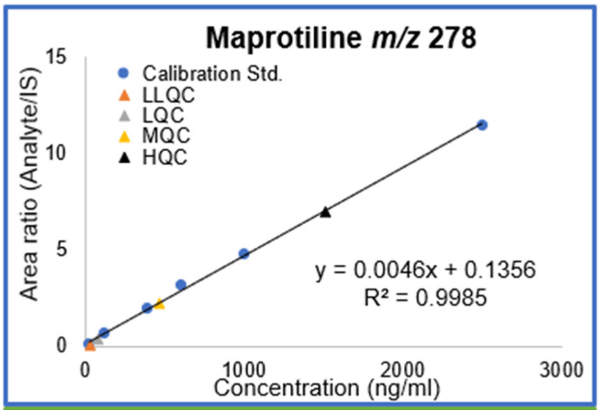
Maprotiline m/z 278
Calibration Std.
LLQC
LQC
MQC
HQC
y = 0.0046x + 0.1356
R² = 0.9985
Area ratio (Analyte/IS)
Concentration (ng/ml)

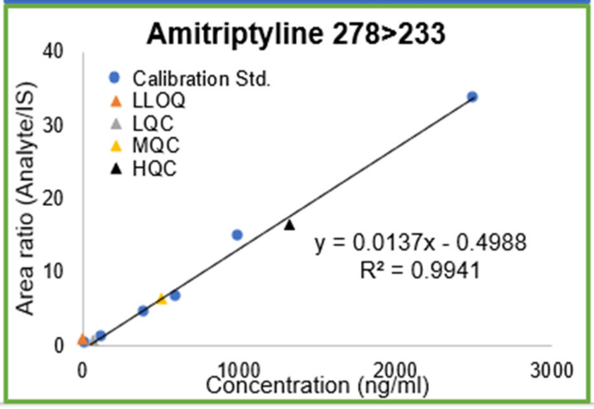
Amitriptyline 278>233
Calibration Std.
LLOQ
LQC
MQC
HQC
y = 0.0137x - 0.4988
R² = 0.9941
Area ratio (Analyte/IS)
Concentration (ng/ml)

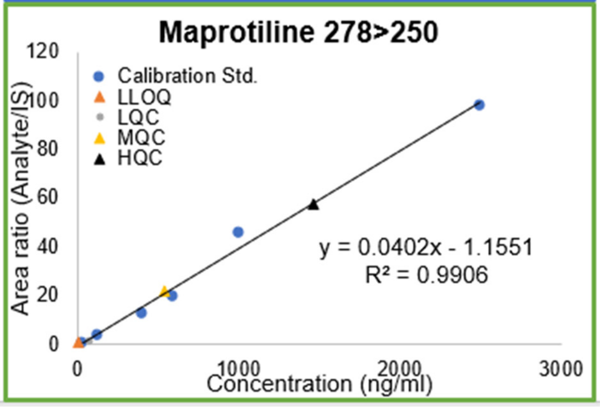
Maprotiline 278>250
Calibration Std.
LLOQ
LQC
MQC
HQC
y = 0.0402x - 1.1551
R² = 0.9906
Area ratio (Analyte/IS)
Concentration (ng/ml)

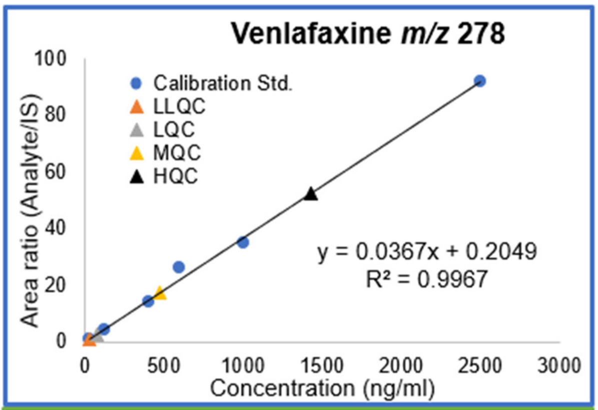
Venlafaxine m/z 278
Calibration Std.
LLQC
LQC
MQC
HQC
y = 0.0367x + 0.2049
R² = 0.9967
Area ratio (Analyte/IS)
Concentration (ng/ml)

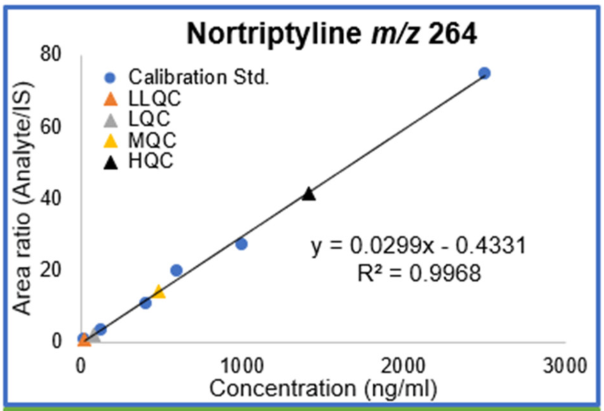
Nortriptyline m/z 264
Calibration Std.
LLQC
LQC
MQC
HQC
y = 0.0299x - 0.4331
R² = 0.9968
Area ratio (Analyte/IS)
Concentration (ng/ml)

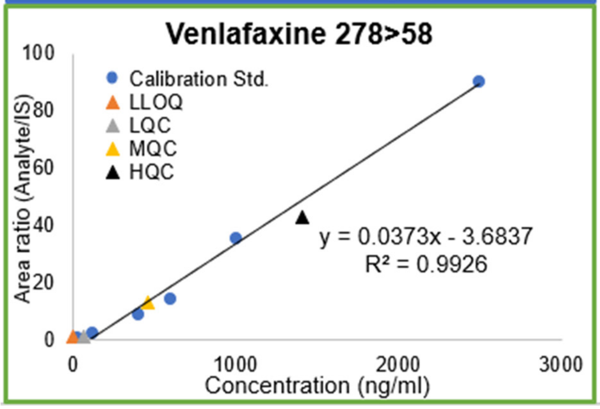
Venlafaxine 278>58
Calibration Std.
LLOQ
LQC
MQC
HQC
y = 0.0373x - 3.6837
R² = 0.9926
Area ratio (Analyte/IS)
Concentration (ng/ml)

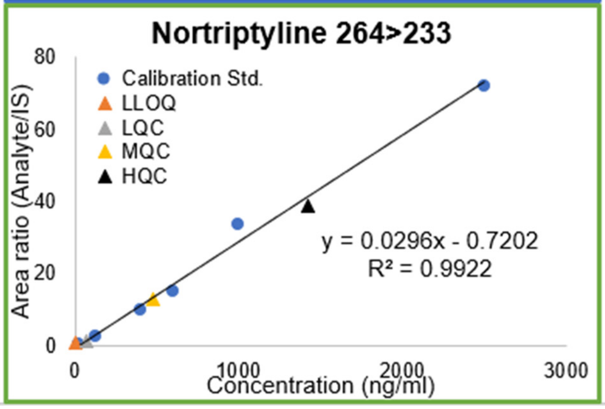
Nortriptyline 264>233
Calibration Std.
LLOQ
LQC
MQC
HQC
y = 0.0296x - 0.7202
R² = 0.9922
Area ratio (Analyte/IS)
Concentration (ng/ml)

Figure S6: Inter-assay accuracy at LLOQ level (25 ng/ml) of spiked antidepressant drugs in plasma samples acquired by LC-MRM/MS and LC-DMS-SIM/MS methods.

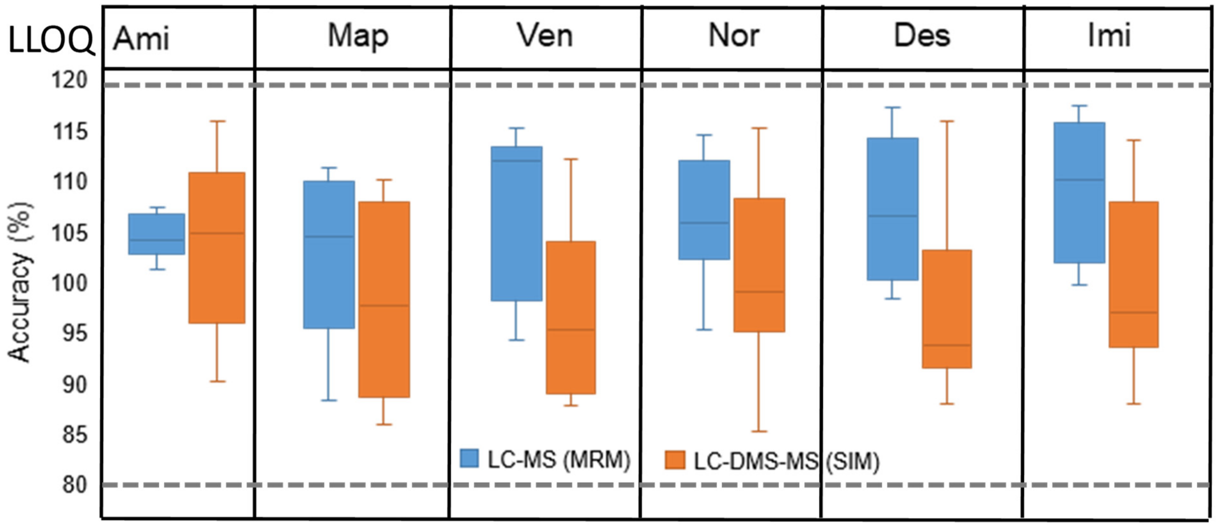

Figure S7: Representative extracted ion chromatograms LC-MRM/MS of human plasma with 400 ng/mL antidepressants drugs (IS 150 ng/mL)

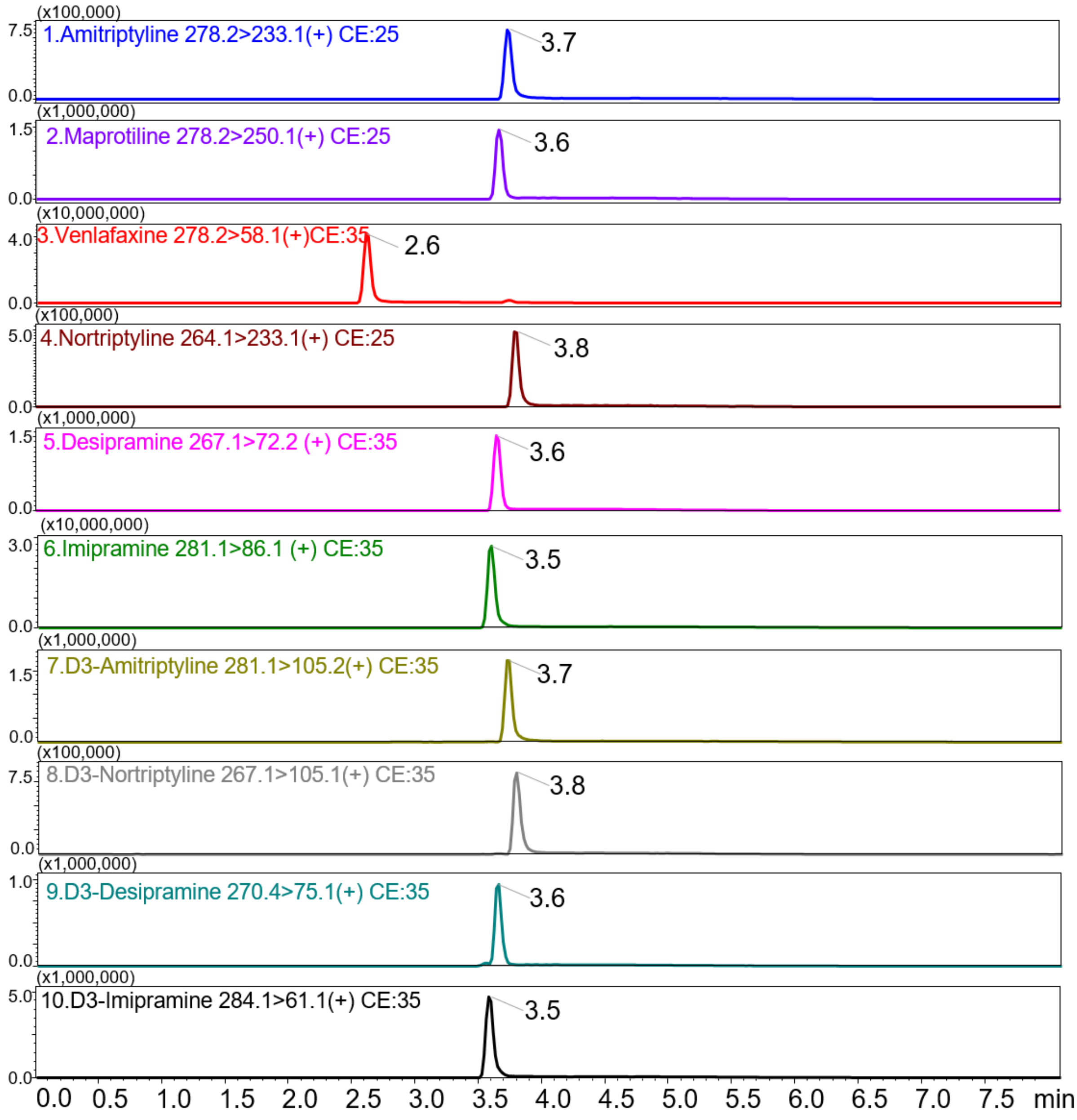

Table S1: MS conditions and RT for LC-DMS-SIM/MS and LC-MRM/MS methods. N/A not applied, IS : Internal Standard

	LC-MRM/MS					LC-DMS-SIM/MS			
Antidepressants	Q1 (m/z)	Q3 (m/z)	CE (V)	RT (min)	IS	RT (min)	IS	CV (V)	DV (V)
Amitriptyline (A)	278.2	233.1	25	3.7	A-D3	0.7	I-D3	6.8	760
		105.2	35						
Maprotiline (M)	278.2	250.1	29	3.6	A-D3	0.7	I-D3	-1.1	760
		117.1	35						
Venlafaxine (V)	278.2	58.1	43	2.6	A-D3	0.7	I-D3	10.4	760
		121.1	25						
Nortriptyline (N)	264.1	233.1	21	3.8	N-D3	0.7	I-D3	-4.9	760
		105.1	35						
Desipramine (D)	267.1	72.2	29	3.6	D-D3	0.7	I-D3	-1.9	760
		193.1	35						
Imipramine (I)	281.1	86.1	25	3.5	I-D3	0.7	I-D3	10.4	760
		58.1	35						
D_3-Amitriptyline (A-D3)	281.1	233.1	25	3.7	N.A.	N.A.	N.A.	N.A.	N.A.
		105.2	35						
D_3-Nortriptyline (N-D3)	267.2	233.1	21	3.8	N.A.	N.A.	N.A.	N.A.	N.A.
		105.1	35						
D_3-Desipramine (D-D3)	270.2	75.1	29	3.6	N.A.	N.A.	N.A.	N.A.	N.A.
		193.1	25						
D_3-Imipramine (I-D3)	284.1	61.1	25	3.5	N.A.	0.7	-	10.6	760
		89.1	35						
